# Cortical Stimulation Paired With Volitional Unimanual Movement Affects Interhemispheric Communication

**DOI:** 10.3389/fnins.2021.782188

**Published:** 2021-12-24

**Authors:** Richy Yun, Andrew R. Bogaard, Andrew G. Richardson, Stavros Zanos, Steve I. Perlmutter, Eberhard E. Fetz

**Affiliations:** ^1^Department of Bioengineering, University of Washington, Seattle, WA, United States; ^2^Washington National Primate Research Center, University of Washington, Seattle, WA, United States; ^3^Department of Physiology and Biophysics, University of Washington, Seattle, WA, United States; ^4^Department of Neurosurgery, University of Pennsylvania, Philadelphia, PA, United States; ^5^Institute of Bioelectronic Medicine, Feinstein Institutes for Medical Research, New York, NY, United States

**Keywords:** alpha coherence, electrical cortical stimulation, interhemispheric inhibition, motor cortex, non-human primate, timing-dependent plasticity

## Abstract

Cortical stimulation (CS) of the motor cortex can cause excitability changes in both hemispheres, showing potential to be a technique for clinical rehabilitation of motor function. However, previous studies that have investigated the effects of delivering CS during movement typically focus on a single hemisphere. On the other hand, studies exploring interhemispheric interactions typically deliver CS at rest. We sought to bridge these two approaches by documenting the consequences of delivering CS to a single motor cortex during different phases of contralateral and ipsilateral limb movement, and simultaneously assessing changes in interactions within and between the hemispheres *via* local field potential (LFP) recordings. Three macaques were trained in a unimanual reaction time (RT) task and implanted with epidural or intracortical electrodes over bilateral motor cortices. During a given session CS was delivered to one hemisphere with respect to movements of either the contralateral or ipsilateral limb. Stimulation delivered before contralateral limb movement onset shortened the contralateral limb RT. In contrast, stimulation delivered after the end of contralateral movement increased contralateral RT but decreased ipsilateral RT. Stimulation delivered before ipsilateral limb movement decreased ipsilateral RT. All other stimulus conditions as well as random stimulation and periodic stimulation did not have consistently significant effects on either limb. Simultaneous LFP recordings from one animal revealed correlations between changes in interhemispheric alpha band coherence and changes in RT, suggesting that alpha activity may be indicative of interhemispheric communication. These results show that changes caused by CS to the functional coupling within and between precentral cortices is contingent on the timing of CS relative to movement.

## Introduction

Cortical stimulation (CS) delivered during different phases of movement has been shown to affect motor function (Pascual-Leone et al., 1992; Bütefisch et al., 2004; Thabit et al., 2010; Edwardson et al., 2015). However, these studies often have limited timing of stimulation and examine one limb and the contralateral hemisphere in isolation. Although limb movements are predominantly driven by the contralateral motor cortex, entirely unilateral movements elicit correlated activity in the ipsilateral hemisphere (Tanji et al., 1988; Donchin et al., 1998; Cardoso et al., 2001). Evidence shows that the motor cortex recruits transcallosal networks that primarily evoke interhemispheric inhibition (IHI) in homologous regions of the contralateral motor cortex (Ferbert et al., 1992; Cincotta and Ziemann, 2008; Hübers et al., 2008).

Interhemispheric inhibition has been directly demonstrated with transcranial magnetic stimulation (TMS) using a paired-pulse paradigm. A test stimulus following a conditioning stimulus of the contralateral hemisphere with a 6–15 ms delay results in depressed motor evoked potentials (MEPs) (Ferbert et al., 1992; Di Lazzaro et al., 1999; Daskalakis et al., 2002). The interstimulus interval (ISI) between the conditioning and test stimulus affects the strength of IHI; ISIs of 3–5 ms can even produce facilitation of the MEPs (Ferbert et al., 1992; Hanajima et al., 2001). Similar to the paired-pulse paradigms, stimulation of the ipsilateral M1 during voluntary movements attenuates the electromyographic (EMG) activity of the moving limb, termed the ipsilateral silent period (iSP) (Ferbert et al., 1992; Cincotta and Ziemann, 2008; Giovannelli et al., 2009). IHI is additionally modulated by various attributes of the motor output, such as the fineness of movements and contraction strength (Perez and Cohen, 2008; Morishita et al., 2012; Kuo et al., 2017). Further findings document complex interactions of IHI with various intracortical inhibitory circuits in the motor cortex (Daskalakis et al., 2002; Chen et al., 2003).

**FIGURE 1:**
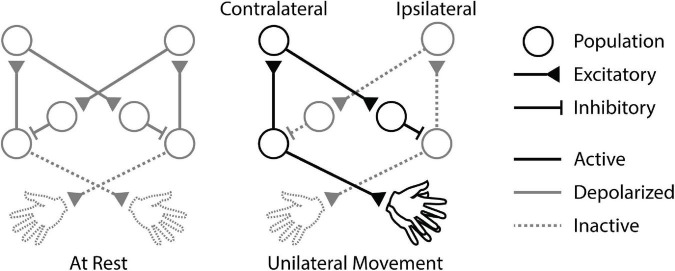
Cortical circuitry during unilateral movement. When the animal is at rest, baseline interhemispheric inhibition (IHI) between the motor cortices is depolarized. Unilateral movement activates the contralateral cortex, triggering IHI to the ipsilateral cortex and disinhibiting itself.

These studies indicate the potential impact of IHI on cortical activity throughout unilateral movements (Figure 1). However, the associated temporal dynamics are not well understood because paired-pulse paradigms are typically performed at rest and iSP experiments typically deliver stimulation during tonic contraction. The effects of stimuli delivered before, during, and after a brief unilateral movement remain to be compared to determine how stimulation and interhemispheric interactions affect preparation and execution of movements. Additionally, the changes caused by stimulation are usually considered on a trial-by-trial basis without regard for possible prolonged effects. Another open question is how long the effects of a single stimulus last.

In addition, despite extensive research on the behavioral effects of IHI in M1, there have been surprisingly few relevant neural recordings. Different frequency bands of local field potentials (LFPs) exhibit unique modulation throughout movement planning, execution, and termination, and are thought to reflect underlying excitatory and inhibitory circuitry (Murthy and Fetz, 1996; Baker et al., 1999; Engel et al., 2001). In particular interest are the alpha band (8–12 Hz) (Hummel et al., 2002; Jensen and Mazaheri, 2010) and the beta band (15–30 Hz) (Murthy and Fetz, 1996; Engel and Fries, 2010; Heinrichs-Graham et al., 2017) which have both been shown to be consistently modulated during visuomotor tasks and associated with top-down processing. Correlated changes in LFP bands and behavior induced by CS would shed light on the dynamics of IHI.

This study probed the temporal dynamics of unimanual movement and the relevance of interhemispheric interactions by delivering CS at different phases of a cued voluntary movement. Monkeys performed a unimanual reaction time (RT) task as we delivered electrical CS to one hemisphere during either contralateral or ipsilateral hand movements; stimuli were timed to arrive during movement preparation, during movement execution, or after movement completion. We tracked the RT of both limbs throughout the experiment and found that RT of the trigger limb (tRT) decreases if the stimulation is delivered before movement, and tRT decreases but the RT of the non-trigger limb (ntRT) increases if the stimulation is delivered after the movement of the contralateral limb. Additionally, we simultaneously recorded LFPs from the motor cortices of both hemispheres and performed coherence analysis to elucidate the neural correlates of interhemispheric communication. Changes in interhemispheric LFP coherence suggest that alpha oscillations may be a signature of interhemispheric communication.

## Materials and Methods

### Subjects and Implants

Experiments were conducted with three male pigtail macaques (*Macaca nemestrina*; Monkeys I, K and U). All procedures conformed to the National Institutes of Health *Guide for the Care and Use of Laboratory Animals* and were approved by the University of Washington Institutional Animal Care and Use Committee.

Monkey I received 8 custom epidural electrodes bilaterally (Supplementary Figure 1A). Epidural electrodes were constructed with 0.01-inch platinum wire insulated with heat-shrink tubing, with a ∼0.5 mm exposed tip. Monkeys K and U were implanted with custom-made electrodes combining epidural and depth electrodes called “dual electrodes” (Supplementary Figure 1B, detailed in Seeman et al., 2017) over left and right sensorimotor cortex (U: 7 electrodes bilaterally, K: 2 electrodes bilaterally; Supplementary Figure 1A). In brief, dual electrodes were constructed using two 0.005-inch bare platinum-iridium (PtIr) wire rods. For each dual electrode, two rods soldered to lead wires were secured in a small piece of polytetrafluoroethylene (PTFE) tubing with silicon glue. The other end of the lead wires was soldered to connectors. The tips of the rods were placed ∼0.5 and 2–2.5 mm from the edge of the PTFE tube such that the shorter rod rests on the surface of the dura and the longer rod reaches layer V of the cortex.

### Implant Surgeries

All surgeries were performed under isoflurane anesthesia and aseptic conditions. For implant of both types of electrodes, an incision was made along the midline of the scalp, and muscle and connective tissue were resected to expose the skull. Titanium skull screws were placed in the skull around the edge of the exposure for electrical grounds and mechanical stability. Small holes for the electrodes were drilled over the sensorimotor cortex with a 1 mm bit using stereotactic coordinates. One electrode was placed with forceps into each hole until resistance was felt between the rod and the dura. Dual electrodes were additionally pushed through the dura and into the cortex until a second resistance was felt between the shorter rod and the dura. After all electrodes for one hemisphere were implanted, a thin coat of dental acrylic (methyl methacrylate) was used to seal the holes and hold the electrodes in place. A titanium casing was then placed over the implant and secured to the skull screws with acrylic. The connectors for the electrodes were cemented to the skull within the casing. Animals received postoperative courses of analgesics and antibiotics.

### Behavior

**FIGURE 2:**
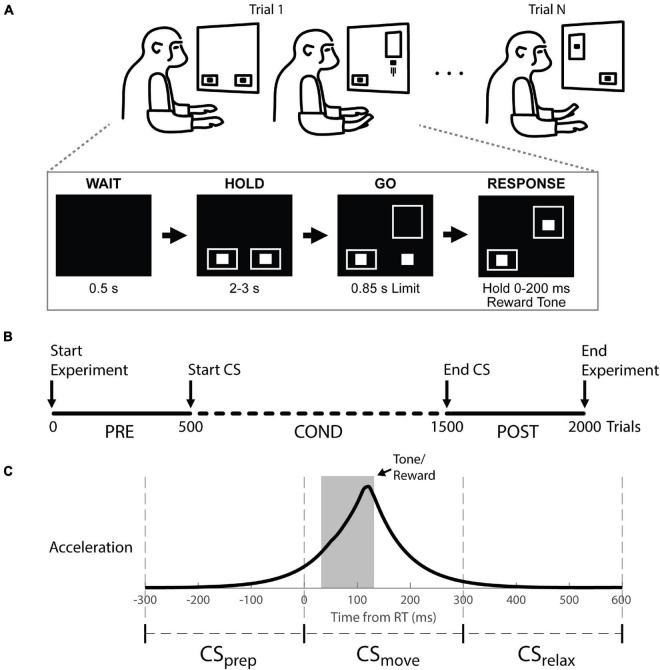
Behavioral task, experimental timeline, and stimulus timing. **(A)** Top: Monkeys were cued to move a cursor into a target box by rapid wrist extension. Trials were randomly selected to be left or right. Bottom: example right hand trial. **(B)** Experimental timeline showing trials before (Pre), during (Cond) and after (Post) cortical stimulation (CS). The task was performed continuously throughout the experiment. **(C)** Stimulus timings are shown with respect to the average accelerometer trace across trials. The stimulus timing was split into three groups relative to movement onset (RT at 0): CS_prep_, CS_move_, and CS_relax_.

**FIGURE 3:**
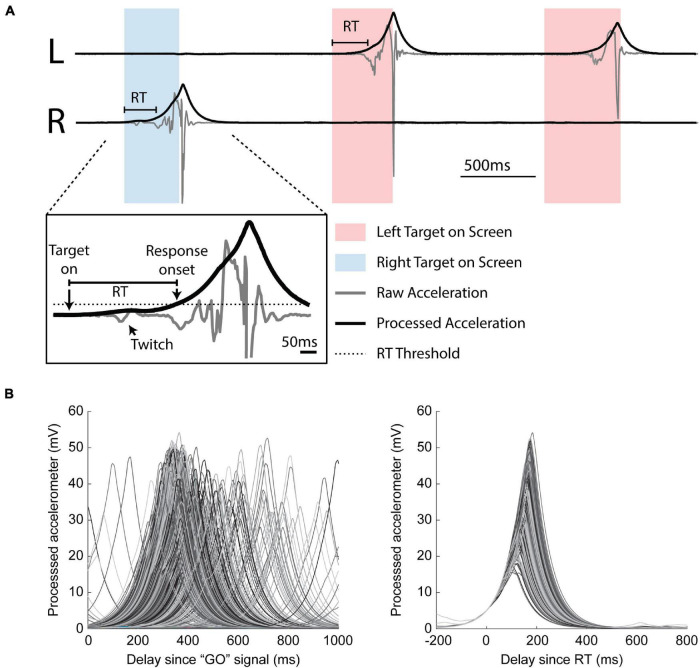
Estimating reaction time. **(A)** Three example trials. Boxes indicate when the monkey was cued to move left (red box) or right (blue box). Responses were required to be unilateral. Inset: identification of reaction time (RT). Raw accelerometer signal (gray) is transformed into a processed signal (black) for RT estimation using a threshold (dotted line). Note the stimulus induced twitch lies below the threshold. **(B)** Aligned processed accelerometer traces. Left: aligned by GO signal. Right: aligned by calculated RT.

During each session, the monkeys were seated in a primate chair facing a monitor (Figure 2A) and 3-axis accelerometers were affixed to the dorsum of each hand. The vertical position of two cursors on the screen were controlled by the z-axis of each accelerometer (rectified and smoothed with a 50 ms boxcar filter). Each trial began with a 0.5 s delay, followed by target boxes at the bottom of the screen to cue the animal to hold at rest. After holding for 2–3 s, one of the target boxes moved up (the “GO” signal) to cue a quick, unilateral wrist extension to drive the associated cursor into the box within 0.85 s. When the cursor was held in the target for 0–200 ms while the other cursor stayed in the start box, an apple smoothie reward was delivered with an audio tone. Trials were randomly selected to be left or right limb and the task was performed throughout the experiment.

### Cortical Stimulation

Each stimulus consisted of a 10-pulse train delivered at 333 Hz. Biphasic pulses were negative leading with a 200 μs phase width. 200 μs phase width pulses have been shown to reliably activate cortical circuitry and have also been used to induce plasticity in the primate cortex (Jackson et al., 2006; Logothetis et al., 2010). CS was bipolar, delivered between two adjacent electrodes for epidural electrodes (Monkey I) or between the surface and intracortical contacts of dual electrodes (Monkeys K and U). Current amplitude was determined daily as the lowest intensity to elicit movement in the contralateral limb as determined by the investigator using the accelerometer trace. The stimulated hemisphere was chosen randomly each session, and CS was triggered by either the contralateral limb (Contralateral CS experiments) or the ipsilateral limb (Ipsilateral CS experiments). The timing of CS for each session was determined randomly before each experiment and kept consistent throughout the session.

For Monkeys I and K, CS was delivered to 100% of the trigger trials during stimulation (e.g., 100% of contralateral trials in Contralateral CS experiments). However, the suprathreshold stimulation completely obscured LFPs in the stimulated hemisphere. To obtain artifact-free LFP recordings during stimulation, CS was delivered to only a random 50% of the trials for Monkey U (e.g., 50% of contralateral trials in Contralateral CS experiments). The data from Monkey U was used for all LFP analysis of the conditioning period (Cond epoch, see section “Experimental Design”).

Control experiments consisted of no stimulation, periodic stimulation, or randomly timed stimulation. There was no difference between baseline left and right limb RT, so all behavioral data was combined as RT of the limb with respect to the stimulated hemisphere. Thus, RTs during Contralateral CS, Ipsilateral CS, and randomly timed experiments are presented as the trigger limb RT (tRT) and non-trigger limb RT (ntRT). Data for Contralateral CS and control experiments are from all three monkeys and Ipsilateral CS experiments are from Monkeys K and U.

### Experimental Design

The timeline of an individual experimental session is depicted in Figure 2B. In each session the initial 500 trials were collected to characterize RT in the absence of CS (Pre epoch). We discarded the first 25 trials to avoid warm-up effects as the monkey settled into the task. After the Pre epoch, CS was initiated and continued for about 1,000 trials (Cond epoch). At the end of conditioning, another 500 trials were collected (Post epoch) to determine the longevity of any induced changes. Each session lasted for 1.5–2.5 h. One session was performed per day to prevent any effects from propagating between sessions. All changes in RT are shown as the difference between the RTs during the Cond or Post epoch and the median RT during the Pre epoch (ΔRT).

Movement onset was determined by threshold crossing of the smoothed rectified acceleration trace (see section “Recordings and Analysis”). The sessions were labeled according to the response phase during which stimuli were delivered relative to movement onset—preparatory phase, CS_prep_: −300–0 ms; during movement, CS_move_: 0–300 ms; after movement, CS_relax_: 300–600 ms (Figure 2C and Supplementary Figure 2). We ensured a delay of 2–3 s between trials such that CS_relax_ could not be construed as CS_prep_ of the next trial.

### Recordings and Analysis

Behavioral data, including cursor position, target presentation time, and raw accelerometer signal, were recorded at a sampling rate of 1 kHz (National Instruments Multifunction I/O Device). During the task, cursor position was driven by accelerometer signals generated by each wrist (Figure 3A). RT was measured offline using the raw accelerometer signal processed in three steps: 1) band-pass filtered between 10 and 150 Hz, 2) rectified, and 3) low pass filtered at 5 Hz. We calculated a movement threshold as 1/6 the peak of the median response over the last 600 ms from trial completion as this ensured we did not detect the stimulus induced movement as movement onset (Supplementary Figure 3). RT was defined as the time between the target presentation (GO signal) and the threshold crossing of the processed accelerometer response (Figure 3A).

For each animal, LFPs were recorded single-ended between each individual electrode and a distant reference from the same hemisphere. Sampling rates were 9.6 kHz for Monkeys U and K, and 2.4 kHz for Monkey I (g.USBamp, Guger Technologies). LFP frequency bands were extracted using a third-order bandpass Butterworth filter (alpha: 8–12 Hz, beta: 15–30 Hz, low gamma: 30–50 Hz). Instantaneous amplitude for each filtered signal was calculated offline as the absolute value of the Hilbert transform. Spectral density and cross-spectral densities were calculated using the multitaper method [Chronux software package (Mitra and Bokil, 2008; Mitra et al., 2018)] with an overlapping moving window of 500 ms width and 25 ms steps. LFP traces in figures are the average across all electrodes in the designated hemisphere.

Magnitude-squared coherence was calculated by:

(1) $$C_{x\,y}=\frac{{\left|P_{x\,y}\,\left(f\right)\right|}^{2}}{P_{x\,x}\,\left(f\right)\,P_{y\,y}\,\left(f\right)}$$

where *C*_*xy*_ is the coherence between *x* and *y*, *P*_*xy*_ (*f*) is the cross-spectral density between *x* and *y*, and *P*_*xx*_ (*f*) and *P*_*yy*_(*f*) are the spectral densities of *x* and *y* respectively. In all coherence analyses, *x* and *y* were signals from electrodes in different hemispheres. Coherence traces in figures are the average of all pairwise combinations of electrodes between the two hemispheres.

To assess whether there was a direction of connectivity in the LFP bands we calculated Granger causality (Granger, 1969). Granger causality uses an autoregressive model to determine whether a signal can predict another signal, which would imply a direction of information transfer between the two signals. We used the Multivariate Granger Causality (MVGC) toolbox (Barnett and Seth, 2014) to perform Granger causality analyses on all pairs of channels. LFPs from each channel were preprocessed with a comb notch filter at 60 Hz to ensure predictability did not arise from noise.

All offline analyses were conducted using custom MATLAB scripts.

### Statistical Analysis

Each animal’s performance fluctuated over time, possibly due to changes in motivation or fatigue. As a result, all statistical tests for RT and LFPs compared the changes in distributions from the no stimulation control experiments (e.g., a difference between the Pre epoch and the Cond epoch in a Contralateral CS experiment was considered significant if it was statistically distinct from the difference between the Pre epoch and the Cond epoch in control sessions). The Wilcoxon rank-sum test was used for all hypothesis testing due to the non-parametric nature of the data.

All box plots show the median and interquartile range. The notch represents:

(2) $$m\pm \frac{1.57\,I\,Q\,R}{\sqrt{n}}$$

where *m* is the median, *IQR* is the interquartile range, and *n* is the number of samples. The full range (whiskers) and outliers have been omitted for clarity.

## Results

### Data

We recorded a total of 152 sessions from three monkeys (I: 67; K: 31; U: 54). The number of experimental conditions per animal is shown in Table 1. Figure 3 shows that our movement-detection algorithm successfully detected RTs in single trials. Variability in the trial-by-trial response is evident in the accelerometer snippets aligned with the GO signal (Figure 3B, left), and the onset of the earliest responses is evident at around 100–200 ms. Aligning the accelerometer traces with RT (Figure 3B, right) demonstrates that the monkeys’ movements were consistent.

**TABLE 1:** Number of experiments by type.

Monkey	Ipsilateral CS	Contralateral CS	No stim	Non-time-locked stim
I	3	41	10	13 (periodic)
K	14	14	3	0
U	20 (50% CS)	22 (50% CS)	4	6 (random)

*Number of experiment types per animal.*

*Monkey I only had 3 Ipsilateral CS experiments and is hence omitted from Ipsilateral CS analyses.*

### Contralateral Cortical Stimulation

**FIGURE 4:**
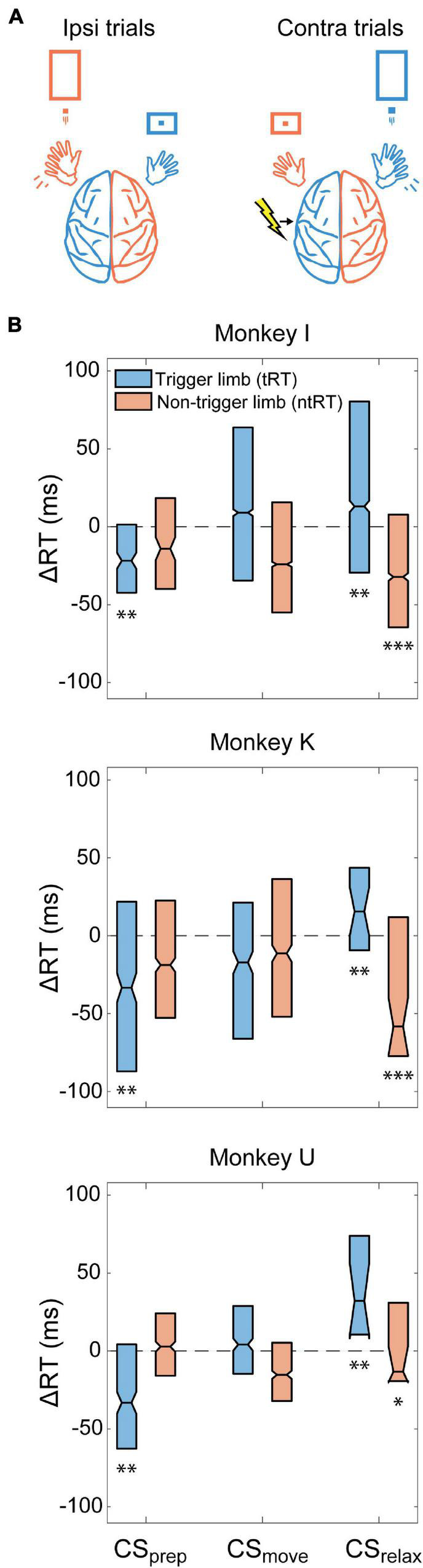
Contralateral CS. **(A)** Example illustration of Contralateral CS and task relationship for left hemispheric stimulation. **(B)** Difference between RTs for trials during the Cond epoch and the median RT of the Pre epoch for each monkey (ΔRT). CS_prep_ significantly decreased tRT in all monkeys. CS_relax_ experiments produced bilateral effects, significantly slowing down tRT and speeding up ntRT. Significance is calculated in comparison with the no stimulation control experiments and only denoted if consistent across all animals (*p < 0.01; **p < 0.001; ***p < 0.0001).

We conducted Contralateral CS experiments in all three monkeys to assess how stimulation of the movement-generating hemisphere affects RTs. Figure 4A depicts an example of behavior and stimulation relationship during the task when CS was triggered with right hand movements. We found that stimulation before movement (CS_prep_, −300 to 0 ms from RT) significantly decreased the trigger limb RT (tRT) in all monkeys but did not significantly affect the non-trigger limb RT (ntRT) (Figure 4B). Surprisingly, stimulation delivered after the movements had concluded (CS_relax_, 300–600 ms from RT) had pronounced effects in subsequent movements associated with both limbs; CS_relax_ significantly slowed tRT but sped up ntRT (Figure 4B). Stimulation during movement execution (CS_move_, 0–300 ms from RT) did not produce a significant or consistent change in either limb’s RT. Changes were denoted to be significantly different from control experiments if they were significant for all three monkeys.

### Ipsilateral Cortical Stimulation

**FIGURE 5:**
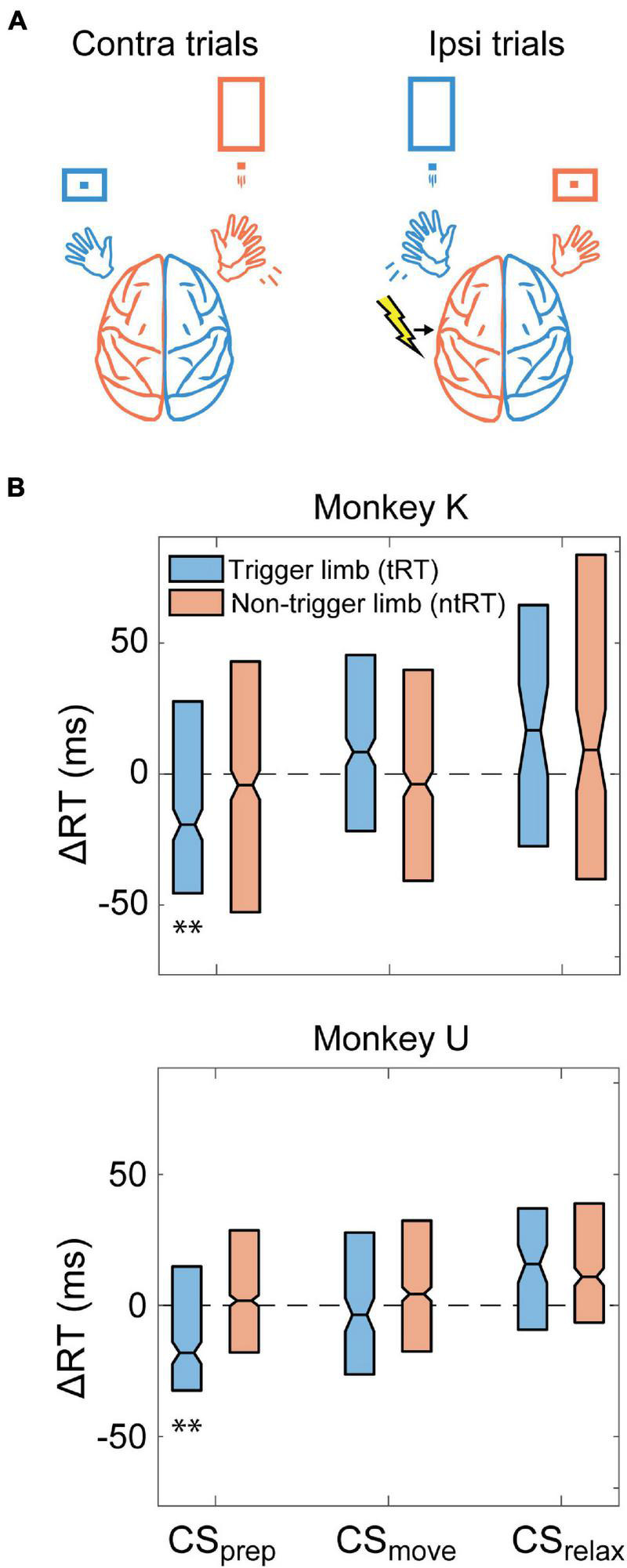
Ipsilateral CS. **(A)** Example illustration of Ipsilateral CS and task relationship for left hemispheric stimulation. **(B)** Difference between RTs for trials during the Cond epoch and the median RT of the Pre epoch for each monkey. CS_prep_ decreased tRT in both monkeys. Significance is calculated in comparison with the no stimulation control experiments and only denoted if consistent across both animals (**p < 0.001).

To further investigate the role of the non-movement generating hemisphere during unilateral movement, we also tested whether CS delivered to the ipsilateral hemisphere could generate changes in RT. These experiments were conducted in Monkeys K and U. Figure 5A depicts an example behavior and stimulation relationship during the task when CS was triggered with left hand movements. Similar to Contralateral CS, we found that CS_prep_ significantly shortened tRT and CS_move_ did not produce a significant or consistent change in RTs of either limb (Figure 5B). CS_relax_ also did not produce any significant effects. Changes were denoted to be significantly different from control experiments if they were significant for both monkeys.

### Persistence of Changes in Reaction Time

**FIGURE 6:**
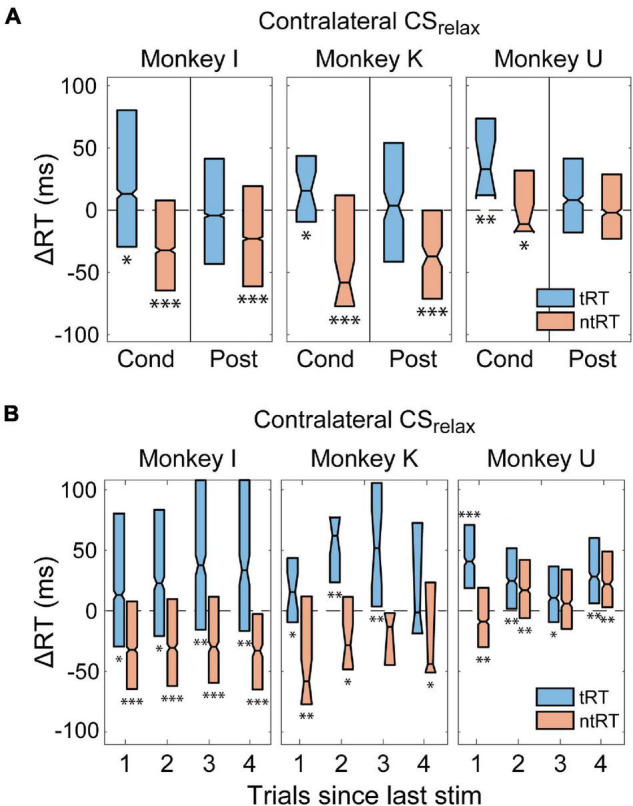
Persistence of changes in RT. **(A)** RT changes of trials ipsilateral to the stimulated hemisphere stayed faster during Post epoch of CS_relax_ experiments in Monkeys I and K. Increases in CRT did not persist (*p < 0.01; **p < 0.001; ***p < 0.001). **(B)** Effects of CS were persistent in trials arriving long after CS was delivered during experiments with consistently timed CS. (*p < 0.01; **p < 0.001; ***p < 0.001).

Previous studies suggested that CS paired with movements or EMG signals can induce lasting directed plasticity (Bütefisch et al., 2004; Thabit et al., 2010; Lucas and Fetz, 2013). Thus, we tracked the changes in RT over time to determine whether the CS we delivered causes long-term changes. Figure 6A shows changes in RT for contra- and ipsilateral trials during the Post epoch for CS_relax_ experiments compared to the Pre epoch. We found that increases in tRT did not persist during the Post epoch but the decrease in ntRT remained consistent in Monkeys I and K. Monkey U did not have a change in the Post epoch, but the effects during the Cond epoch were smaller compared to the other two monkeys. All other stimulus conditions did not have a significant or consistent effect during the Post epoch.

In addition, we analyzed RT of trials without stimulation following a trial with stimulation to document the immediate duration of effects produced by CS. For Monkeys I and K there were no such trials for tRT during Contralateral CS_prep_ and tRT during Ipsilateral CS_prep_ since these trials were always stimulated. The stimulation for CS_relax_, however, arrives after the completion of behavior thus providing us with trials not directly affected by the stimulus. Figure 6B shows that the increase in tRT and decrease in ntRT induced by Contralateral CS_relax_ were just as evident in trials that occurred up to four trials after the most recent stimulation in Monkey I, and at least 1 trial after stimulation for Monkeys K and U.

### Control Experiments

**FIGURE 7:**
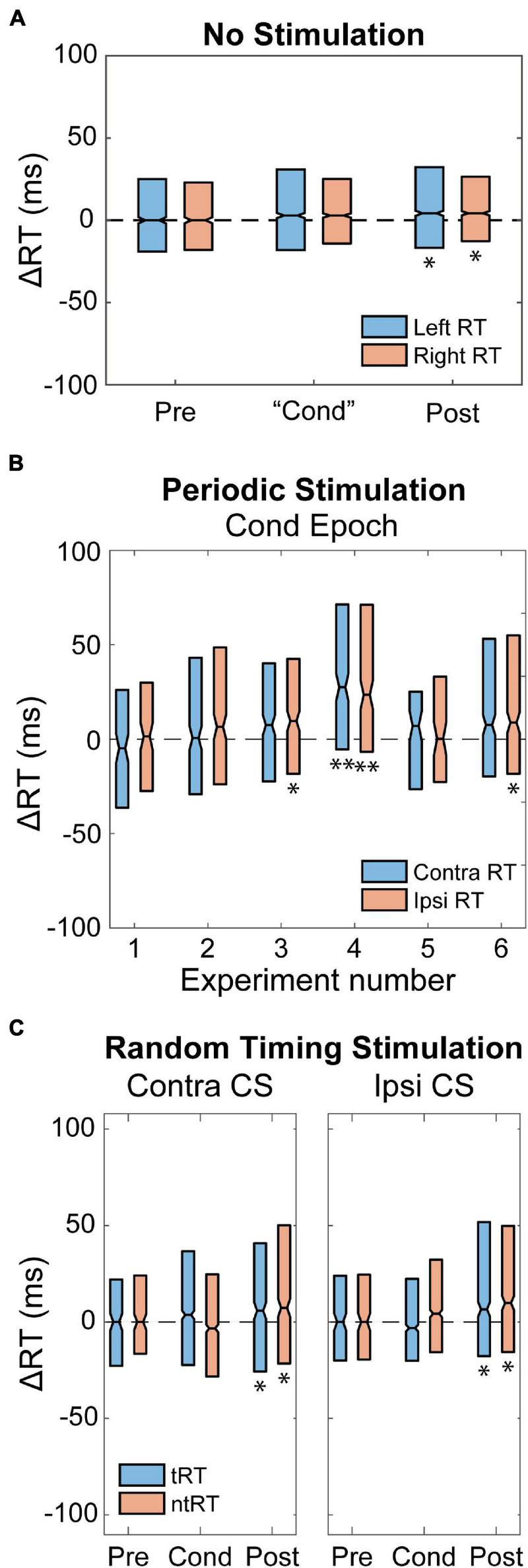
Control Experiments. **(A)** RT relative to the Pre epoch when delivering no stimulation during the “Cond” epoch. The “Cond” epoch was determined using the number of trials. RT slows over time for both limbs and becomes significant by the Post epoch. There was no significant difference between the two limbs. Significance is compared to zero (*p < 0.01). **(B)** Periodic stimulation delivered to one hemisphere did not have consistent changes or produce differential effects in RT associated with either hand. Plots show six different experiments with Monkey I and corresponding change in RT. Contra and Ipsi RT are with respect to the stimulated hemisphere. Significance is compared to zero (*p < 0.01; **p < 0.001). **(C)** Random stimulus timing delivered to both the movement generating (Contra CS) and non-movement generating (Ipsi CS) hemisphere. There is greater variability in RT compared to the no stimulation condition, but median changes were similar. Significance is compared to zero (*p < 0.01).

To control for changes in natural behavior over time we first tracked the animal’s performance without delivering any CS. RT in both limbs slowed over time, becoming significantly slower during the Post epoch compared to the Pre epoch. However, there was no difference between the limbs, which suggests a general behavioral change rather than a specific limb or hemisphere change (Figure 7A). To test whether stimulation itself caused any changes, we performed experiments with open-loop periodic stimulation at 0.1–0.2 Hz. Figure 7B shows that periodic stimulation delivered to one hemisphere during the Cond epoch resulted in large variability, but did not produce consistent changes in either RTs. Periodic stimulation also failed to produce any differential changes between the RTs.

We also performed control experiments to test whether it was necessary that CS was consistently timed to movements over time. Figure 7C shows results from Contralateral and Ipsilateral CS experiments in which stimulation for each trial was randomly delivered between −100 and 800 ms relative to the GO signal. These controls showed similar changes compared to the no stimulation condition (increasing RT during later epochs) for both Contralateral and Ipsilateral CS with greater variability.

### Changes in Cortical Local Field Potentials With Movement

To examine bilateral cortical activation during unilateral movements and changes with stimulation, we recorded local field potentials (LFPs) of both hemispheres from each animal throughout the experiment. Movement-related LFP data is from all three animals and LFP data during the Cond epoch is from Monkey U. Any trials with CS were discarded due to the stimulus artifact obscuring LFPs; as the changes in RT persisted several trials beyond the stimulated trial, the trials between stimulation reflects the effects of CS.

**FIGURE 8:**
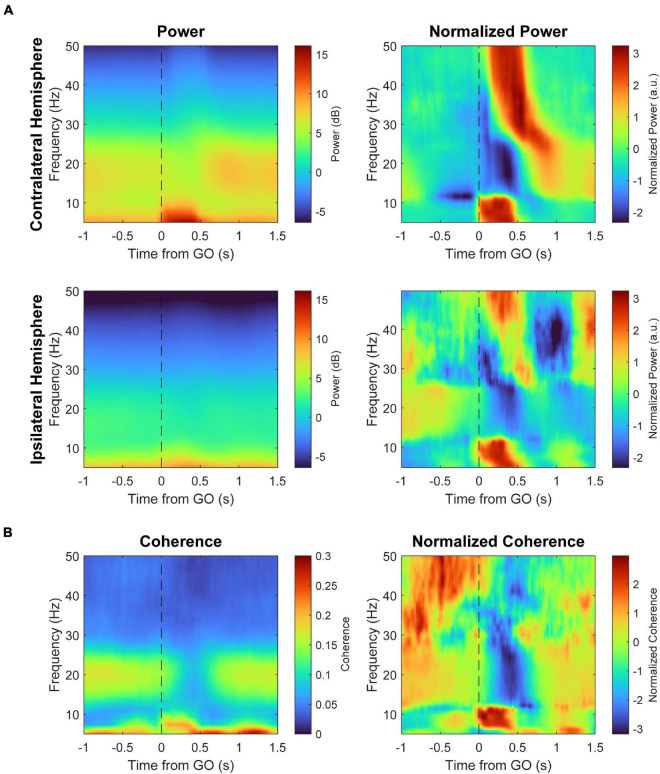
Spectral power and coherence during a trial. **(A)** Spectral power during behavior with respect to the GO signal of the contralateral (top) and ipsilateral hemispheres (bottom) during a Pre epoch, averaged across all three monkeys. The right panels show the spectra normalized across time for each frequency (z-score) to highlight frequency-specific changes over time. **(B)** Coherence between the two hemispheres with respect to the GO signal. The right panel shows coherence normalized across time for each frequency.

**FIGURE 9:**
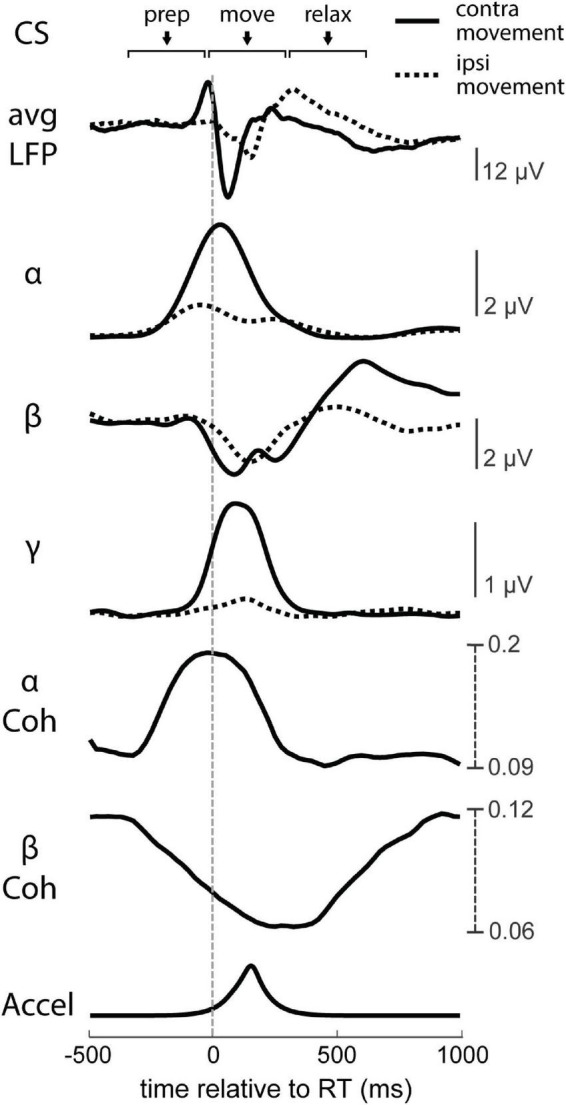
Neural dynamics of movement. RT-averaged raw LFP, instantaneous alpha (α), beta (β), and low gamma (γ) intrahemispheric amplitudes, interhemispheric α and β coherence (Coh), and processed accelerometer signal (Accel). Solid lines depict the contralateral hemisphere’s trial-triggered averages, and dashed lines depict the ipsilateral hemisphere’s trial-triggered averages.

LFPs in primary motor cortex (M1) are consistently modulated with respect to movement in a frequency dependent manner (Figure 8). The raw LFP of each hemisphere around the estimated RT is complex and multiphasic, as shown in Figure 9. We separated the LFPs into different frequency bands (alpha 8–12 Hz, beta 15–30 Hz, and gamma 30–50 Hz) to document the modulation of each band (Figure 9). As commonly reported in intracortical recordings, we observed an increase in alpha and gamma amplitude during movement and a decrease in beta amplitude (Buzsáki and Wang, 2012; Canolty et al., 2012). These LFP band dynamics also reflect MEG recordings in human sensorimotor cortex during cued movements; especially notable are the desynchronization of the beta band during movement onset and its subsequent rebound after movement termination (Cheyne, 2013; Bardouille and Bailey, 2019). Ipsilateral movements generated similar features but with lower amplitude compared to contralateral movements.

We also calculated the change in magnitude-squared coherence of all bilateral pair-wise combinations of sites between the two hemispheres (Figure 9). Alpha coherence peaks at the onset of movement, similar to alpha amplitude. Beta coherence drops during movement, likely due to desynchronization, although the change begins during movement preparation rather than movement onset. Gamma band coherence was inconsistent across trials and experiments, as gamma likely reflects much more local activity (Fries et al., 2007; Buzsáki and Wang, 2012; Buzsáki et al., 2012), and was therefore omitted from coherence analyses.

**FIGURE 10:**
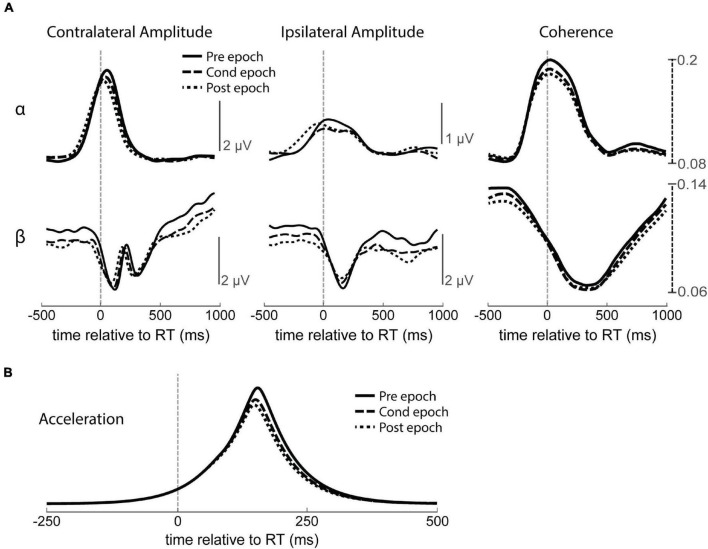
Change in amplitude and coherence over time with no stimulation. **(A)** RT-aligned alpha (α, top) and beta (β, bottom) intrahemispheric amplitude and interhemispheric coherence for all three epochs of no stimulation control experiments. Contralateral and ipsilateral refers to the hemisphere relative to movement. **(B)** Accelerometer traces showing larger movements during the Pre epoch compared to the Cond or Post epochs during no stimulation control experiments.

To document possible changes in LFPs unrelated to CS over the course of a session, we first tracked how these measures change throughout no-stimulation control experiments to document confounding factors such as motivation, fatigue, or attention (Figure 10A). The average amplitude of each frequency band was significantly higher during the Pre epoch, perhaps because the animal performed more exaggerated movements before learning to efficiently move the minimal amount for the task, as well as a possible drop in motivation over time (Figure 10B). Alpha and beta coherence were also higher in the Pre epoch, also possibly related to the larger movements. To account for these changes, all following LFP analyses are performed with respect to the baseline changes observed in control experiments.

### Cortical Stimulation Modulates Interhemispheric Alpha Coherence

We tracked changes in amplitudes and coherence of alpha and beta band LFP to determine neural correlates of interhemispheric communication. The instantaneous amplitudes of alpha and beta band LFP during contralateral or ipsilateral trials in CS experiments are shown in Supplementary Figures 4, 5. A large change observed in LFP bands was the decrease in post-movement beta rebound (PMBR), thought to indicate motor termination (Pfurtscheller et al., 1996; Heinrichs-Graham et al., 2017). However, the changes were consistent across stimulus conditions and PMBR has been shown to decrease with lower force, lower rate of force development, and slower termination of movement (Fry et al., 2016; Heinrichs-Graham et al., 2017). Therefore, the changes in PMBR were likely due to the animal’s movements becoming smaller over time as they became more accurate and fatigued as shown in Figure 10B. Other than the PMBR, there were no clear or consistent differences in LFP band amplitudes during any CS condition compared to control experiments. Ipsilateral CS experiments also showed no significant differences in instantaneous amplitudes of alpha and beta band LFP.

**FIGURE 11:**
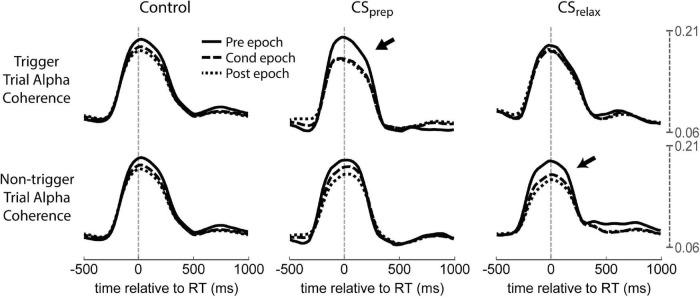
Coherence in Contralateral CS experiments. Coherence during each experimental epoch for CS_prep_ and CS_relax_. Contralateral and ipsilateral refers to limb movement relative to the stimulated hemisphere. Notice the large decrease in contralateral alpha coherence during CS_prep_ and ipsilateral alpha during CS_relax_ (arrows); both RTs decreased during stimulation (Figure 3). CS_move_ did not produce any significant changes. See Supplementary Figure 5 for both alpha and beta coherence for all Contralateral CS conditions.

**FIGURE 12:**
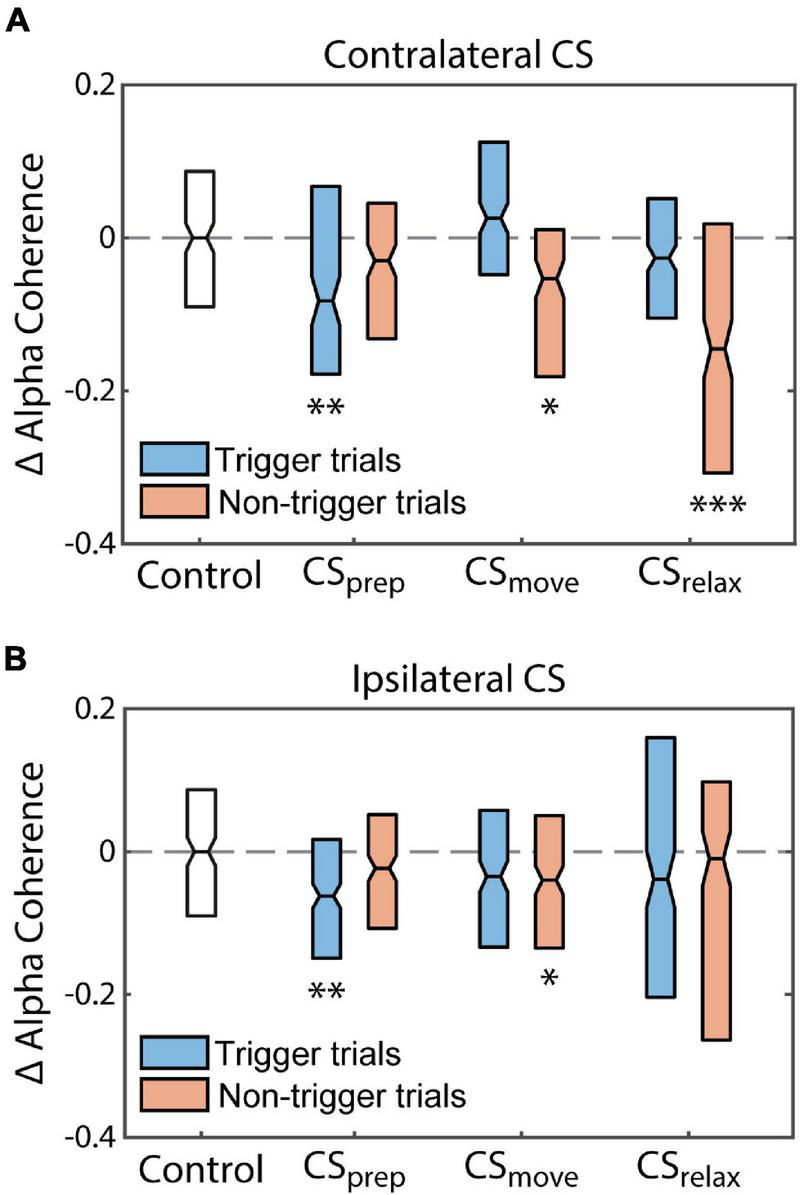
Alpha coherence reflects changes in RT. Normalized changes in the integrated interhemispheric alpha coherence peak from the Pre epoch to the Cond epoch (difference between the coherence in the Pre and Cond epochs divided by the coherence in the Pre epoch). The changes in alpha coherence reflect the changes in Monkey U’s RT induced by each CS timing for **(A)** Contralateral CS and **(B)** Ipsilateral CS. Statistical testing compares the changes to the control experiment. (*p < 0.01, **p < 0.001, ***p < 0.0001).

Beta coherence between hemispheres also did not show any consistent changes, but alpha coherence had clear differences from the control (Supplementary Figures 6, 7). Alpha coherence during trigger trials of Contralateral CS_prep_ was noticeably diminished during the Cond epoch as well as during non-trigger trials of CS_relax_ (Figure 11). To quantify their differences, we integrated alpha coherence in a window of −200 ms to 200 ms from RT to capture the peak amplitude. The decreases in alpha coherence during trigger trials of CS_prep_ and non-trigger trials of CS_relax_ were statistically significant, possibly related to the decrease in RT during those conditions (Figure 12A). Ipsilateral CS experiments also had a significant decrease in alpha coherence that was associated with a decrease in tRT during CS_prep_ (Figure 12B).

There were statistically significant changes in alpha coherence during CS_move_—a decrease during non-trigger trials of Contralateral CS experiments and a decrease during non-trigger trials of Ipsilateral CS experiments—that were not reflected in their respective RT. However, these changes had a relatively high *p*-value, and thus could be due to single-animal variability. Comparisons between the change in alpha coherence and change in RT for all experimental conditions and trial types with a significant change in alpha coherence did not reveal a significant correlation (Supplementary Figure 8).

As IHI is directed from one hemisphere to another, we additionally explored pairwise Granger causality using the MVGC toolbox (Barnett and Seth, 2014). For baseline analysis we used the same window as the alpha coherence peak (−200 to 200 ms from RT) in trials within the Pre epoch during all conditioning experiments for all channel pairs (Supplementary Figure 9). Only 3 out of 32 channels had predictability toward more than one channel on the other hemisphere, and significantly so (*p* < 0.05) in less than 10 out of 42 experiments. In contrast, at least 27 out of 32 channels had significant predictability (*p* < 0.05) within the same hemisphere for at least 10 experiments. We did not pursue Granger causality any further due to the lack of consistent directionality between hemispheres.

## Discussion

**FIGURE 13:**
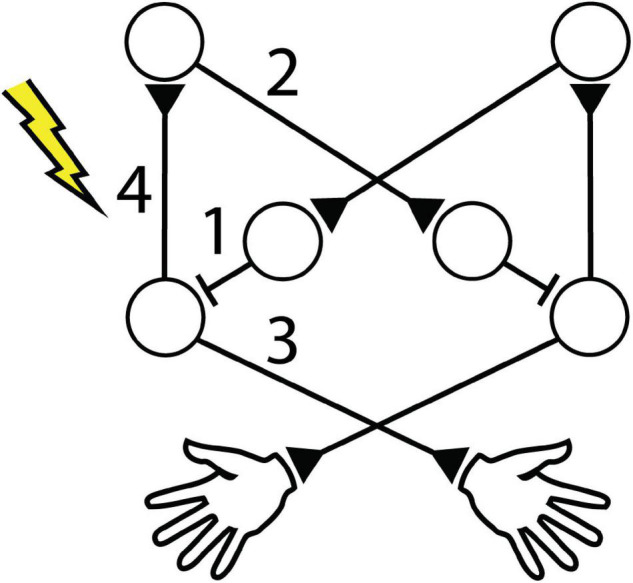
Cortical circuitry affected by stimulation. Intra- and interhemispheric circuitry involved in unilateral movement. Numbers identify proposed affected connections with left hemispheric stimulation for (1) decrease in tRT during Contralateral CS_prep_, (2) decrease in tRT during Ipsilateral CS_prep_, (3) increase in tRT after Contralateral CS_relax_, and (4) decrease in ntRT after Contralateral CS_relax_.

Our main finding was that suprathreshold CS delivered to one motor cortex can affect the RT of both limbs, depending on the timing of CS relative to voluntary unilateral movement. We observed that CS can cause distinct changes in RT: 1) Contralateral CS_prep_ decreased tRT, 2) Ipsilateral CS_prep_ also decreased tRT, and 3) Contralateral CS_relax_ increased tRT but 4) decreased ntRT. Figure 13 shows proposed mechanistic changes induced by each stimulus condition. These changes persisted for at least 4 trials (∼10 s) after the most recent stimulus. The most durable change was the decrease in ntRT following Contralateral CS_relax_, which remained significant during the ∼20-min Post epoch, highlighting the significance of the ipsilateral hemisphere to unilateral movement. The lack of consistent changes in periodic or random stimulation control experiments suggests that changes in RT reflect the effect of consistently timing stimulation to movement throughout the Cond epoch.

We simultaneously recorded cortical LFPs to examine neural dynamics related to movement and how they may be modulated by CS. In control experiments without CS, trial-averaged LFP band amplitude and interhemispheric coherence decreased over time, consistent with the natural increase in RT, perhaps due to increased efficiency in movements or a decrease in motivation. In both contralateral and ipsilateral CS experiments there was a significant change in alpha coherence between the hemispheres that was strongly correlated with decreases in RT, suggesting that alpha may be indicative of IHI.

### Stimulation Delivered Before Contralateral Movement Decreased Contralateral Limb Reaction Time

The suprathreshold stimulation used in our study induces a physical twitch in the animal’s contralateral hand. This movement can be processed as a tactile stimulus, and, since response to tactile stimuli is faster than response to visual stimuli, it may thus have led to a faster RT (Chan and Ng, 2012; Godlove et al., 2014). However, this is unlikely as studies have shown response to electrical microstimulation of the cortex to be significantly slower than response to tactile stimulation in both humans and non-human primates (Godlove et al., 2014; Caldwell et al., 2019).

Suprathreshold stimulus induced movement can also activate cortico-spinal reafferent sensorimotor loops that could lead to changes in RT. Thabit et al. (2010) similarly showed a reduction in contralateral limb RT when suprathreshold TMS was delivered to the motor cortex, but also showed that the F-wave in the corresponding muscle generated by stimulation delivered to the peripheral nerve did not change. Thus, our results are also likely due to changes in cortical excitability rather than changes in the spinal level.

The changes in cortical excitability could be induced by Hebbian-like potentiation. Activation of the motor cortex immediately before activation of the corresponding periphery has been shown to induce long-term potentiation (LTP) in paired-association stimulation (PAS) studies (Wolters et al., 2003; Suppa et al., 2017). Thabit et al. (2010) also showed that the motor-evoked potential (MEP) increased with the reduction in RT. However, such a broad increase in excitability of the cortex would suggest a simultaneous change in IHI, and thus the ipsilateral limb RT, which was not observed. We propose that the stimulation interrupted the inhibitory network responsible for mediating IHI from the contralateral hemisphere (Figures 13, 1). IHI is highest immediately before movement onset (Duque et al., 2007; Beaulé et al., 2012), and electrical CS has been shown to interrupt cortico-cortical signaling (Logothetis et al., 2010; Griffin et al., 2011). As a result, CS could lead to the disinhibition of the cortex and subsequent faster RT. Additional experiments with subthreshold stimulation could potentially confirm these mechanisms.

### Stimulation Delivered Before Ipsilateral Movement Decreased Ipsilateral Limb Reaction Time

Similar to stimulation delivered before contralateral movement, the reduction in ipsilateral RT cannot be attributed to the animal responding to the induced twitch. In addition, reafferent sensorimotor loops do not play a role as the changes were observed in the ipsilateral limb. As the major pathway between the motor cortex and the ipsilateral limb is the transcallosal inhibitory pathway, the stimulation most likely interrupted the ongoing IHI from the stimulated hemisphere to the contralateral hemisphere, thus disinhibiting the contralateral cortex and speeding up the ipsilateral limb RT (Figures 13, 2).

### Stimulation Delivered After Contralateral Movement Increased Contralateral Limb Reaction Time but Decreased Ipsilateral Limb Reaction Time

Paired associative stimulation (PAS) studies have shown that stimulation of the periphery before stimulation of the cortex can induce long-term depression (LTD), thought to be mediated *via* Hebbian-like mechanisms (Wolters et al., 2003; Suppa et al., 2017). Thabit et al. (2010) delivered suprathreshold TMS to the motor cortex after unilateral voluntary movement of the contralateral limb which decreased subsequent MEP amplitudes, also likely through Hebbian-like LTD. Our result showing that Contralateral CS_relax_ increased contralateral RT corroborates these studies—stimulation of the movement-generating hemisphere following voluntary movement increased the RT, likely due to decreased excitability of the stimulated cortex (Figures 13, 3). In addition, during Contralateral CS_relax_ we observed a simultaneous decrease in ipsilateral RT, possibly due to the decreased excitability leading to disinhibition of IHI (Figures 13, 4). Ipsilateral CS_relax_ did not change either RTs, further highlighting the significance of timing CS to specific limb movements.

### Alpha Band Local Field Potential and Interhemispheric Inhibition

Alpha activity has been shown to be inversely related to attention during visuomotor tasks (Klimesch et al., 2007; Jensen and Mazaheri, 2010; Rilk et al., 2011) and is commonly thought of as a resting rhythm. Alpha coherence has similarly been demonstrated to be a measure of reduced cortical activation. When measured during applications of repetitive TMS (rTMS), low-frequency rTMS was shown to induce inhibition and high-frequency stimulation to induce excitation, with corresponding increases and decreases in intra- and interhemispheric alpha coherence (Strens et al., 2002; Thut and Pascual-Leone, 2010). Furthermore, one study applied rTMS in an IHI experiment using a paired-pulse paradigm (Gilio et al., 2003). They demonstrated a decrease in IHI from the stimulated to the non-stimulated hemisphere during delivery of low-frequency rTMS, likely due to disinhibition, though they did not report on relevant LFP measures.

During volitional movement, stimulus-induced IHI of the movement-generating hemisphere has been shown to be highest just before movement but released at movement onset (Duque et al., 2007; Beaulé et al., 2012). The temporal dynamics of interhemispheric alpha coherence in our experiments match these findings; coherence began to rise around the Go signal, reached a peak at movement onset, and decayed during movement execution (Figure 9). Changes in RT that were possibly from modulations of IHI (tRT during Contralateral CS_prep_, tRT during Ipsilateral CS_prep_, and ntRT during Contralateral CS_relax_) were associated with corresponding decreases in their alpha coherence magnitude. The increase in tRT during Contralateral CS_relax_ that is likely due to LTD did not have a significant corresponding change in alpha coherence.

Variations in coherence can often be epiphenomena of changes in overt oscillations (Srinath and Ray, 2014; Buzsáki and Schomburg, 2015). We saw no significant changes in alpha amplitude. Besides changes in amplitude, correlation in amplitude can also greatly affect coherence values (Srinath and Ray, 2014), but we saw very low correlation coefficients (around 0.05) of alpha amplitudes between the hemispheres during movement. Thus, alpha coherence is likely a reflection of changes in synchrony, illustrating interhemispheric alpha coherence as a potential measure of IHI. However, our LFP results during the Cond epoch are drawn from a single animal and further studies are warranted to confirm these findings.

In addition, we explored Granger causality using the MVGC toolbox (Barnett and Seth, 2014). Although we expected signals reflecting IHI to show significant directionality, Granger causality did not reveal any consistent relationships between hemispheres. This may be due to the fact that the time course of IHI is highly volatile, as evidenced by the different changes caused by different ISIs during paired stimulation experiments (Ferbert et al., 1992; Hanajima et al., 2001). Variations within the 400 ms window we tested may have caused difficulties in assessing significant directionality. Another possible explanation is that the frequency range reflecting IHI may be very limited as the only consistent changes in coherence we observed was in the alpha band.

### Clinical Relevance

Studies of stroke patients have found increased inhibition in the lesioned hemisphere, most likely due to compensatory processes that increase activity in the contralesional hemisphere leading to stronger IHI (Liepert et al., 2000; Murase et al., 2004; Lewis and Perreault, 2007). The mechanisms underlying this “IHI imbalance” has been found to predominantly reside in cortical circuitry rather than subcortical structures or pathways (Murase et al., 2004). IHI has also been shown to be especially modulated by movement in stroke patients (Boddington and Reynolds, 2017).

As a result, efforts toward motor recovery have been focused on reducing IHI using rTMS and transcranial direct current stimulation (tDCS) often used in conjunction with rehabilitation (Boddington and Reynolds, 2017). However, the optimal stimulation paradigms for greatest reduction of IHI and most effective recovery is still undetermined. Our experiments suggest that stimulation of the unaffected hemisphere delivered after voluntary movement of the contralesional limb may depress the cortex, thus disinhibiting the lesioned hemisphere for a potentially more effective motor rehabilitation paradigm.

## Conclusion

In conclusion, electrical cortical stimulation delivered to one motor cortex during a unimanual task affected behavior dependent on the timing of stimulation relative to movement. Specifically, stimulation delivered before the contralateral or ipsilateral limb movement sped up the corresponding limb in subsequent trials, likely due to the disruption of ongoing IHI. Stimulation delivered after the contralateral limb movement slowed down the contralateral limb due to STDP-like LTD but sped up the ipsilateral limb as the reduced excitability of the stimulated hemisphere led to the disinhibition of IHI. LFP analyses revealed decreases in interhemispheric alpha coherence during faster reaction times due to decreased IHI, highlighting alpha as a possible measure of interhemispheric communication.

## Data Availability Statement

The raw data supporting the conclusions of this article will be made available by the authors, without undue reservation.

## Ethics Statement

The animal study was reviewed and approved by University of Washington Institutional Animal Care and Use Committee.

## Author Contributions

RY and AB planned and carried out experiments and wrote the manuscript. RY analyzed the data. AR conceived of the original idea with one hemisphere. RY, AB, and SZ extended the project to include interhemispheric interactions. All authors discussed the results, provided feedback, and contributed to the final manuscript.

## Conflict of Interest

The authors declare that the research was conducted in the absence of any commercial or financial relationships that could be construed as a potential conflict of interest.

## Publisher’s Note

All claims expressed in this article are solely those of the authors and do not necessarily represent those of their affiliated organizations, or those of the publisher, the editors and the reviewers. Any product that may be evaluated in this article, or claim that may be made by its manufacturer, is not guaranteed or endorsed by the publisher.
