# Supplementary material for: Cortical Stimulation Paired With Volitional Unimanual Movement Affects Interhemispheric Communication
Source: Front Neurosci. 2021 Dec 24;15:782188. doi: 10.3389/fnins.2021.782188 (PMC8739774; doi:10.3389/fnins.2021.782188)

Supplementary Figure 1. Implant diagrams

(A) Locations of electrodes for each monkey. Electrode locations reflect positions inferred by stereotactic coordinates (3mm spaced grids) at time of implant. **(B)** Diagrams of the custom-made epidural and dual electrodes.

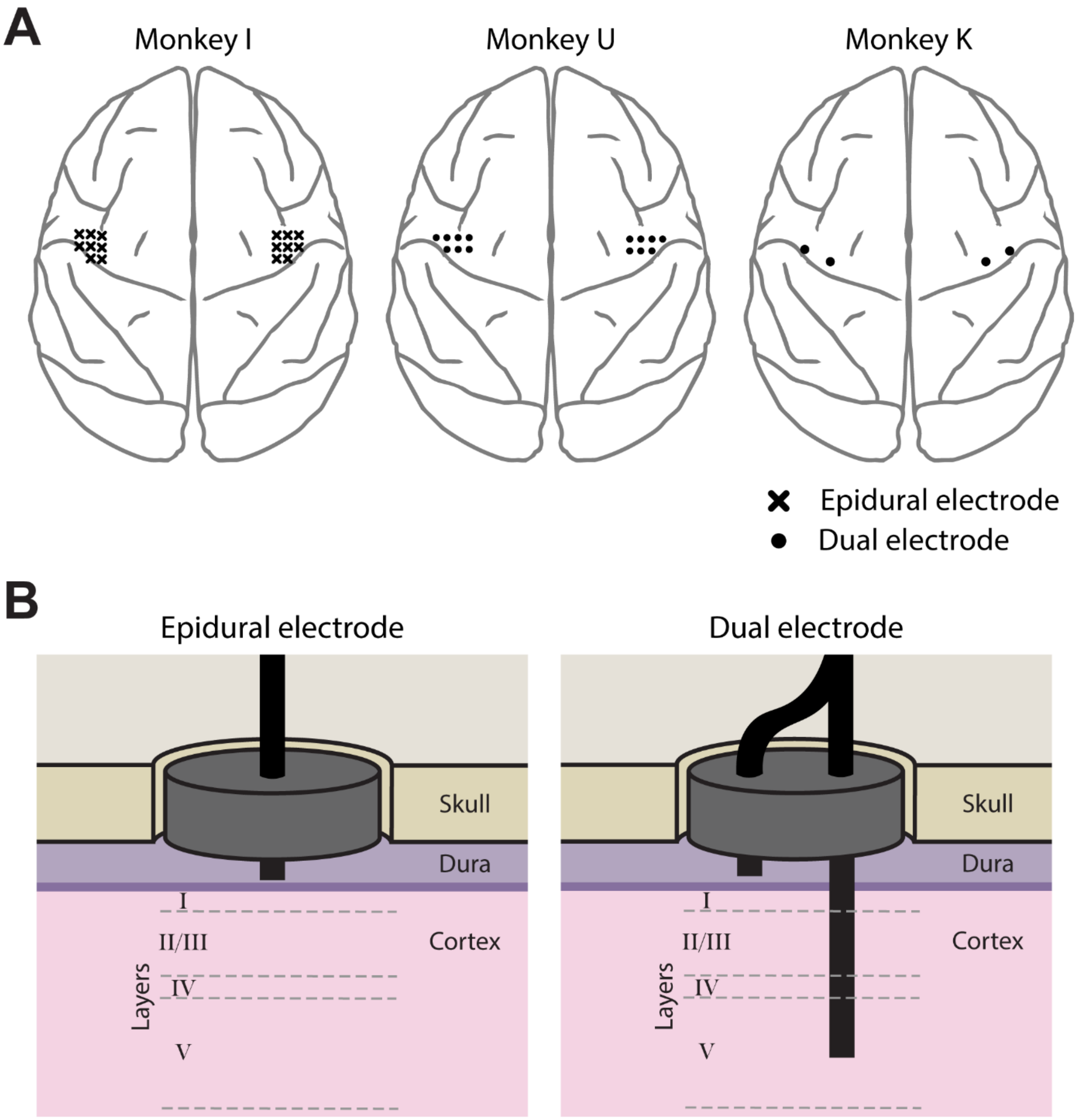

Supplementary Figure 2. Distribution of CS timings

Timing of CS relative to RT for each experiment. The total experiment counts are: 38 in CS_{prep}, 55 in CS_{move}, and 23 in CS_{relax}.

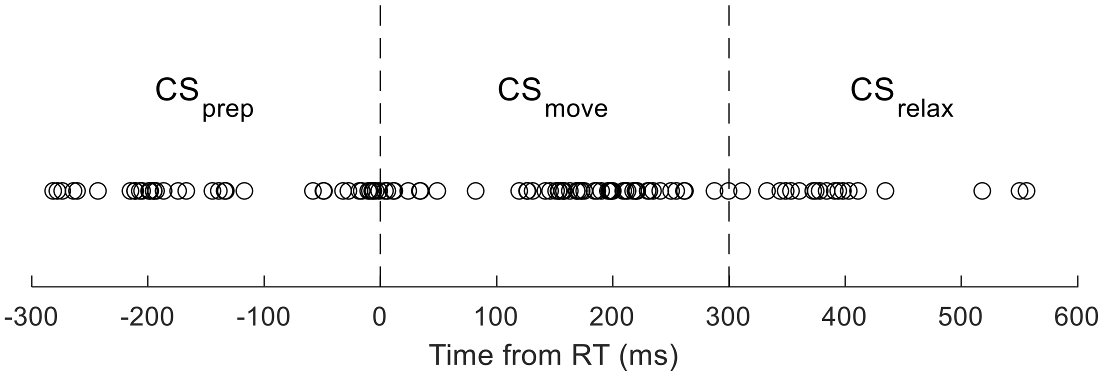

Supplementary Figure 3. **Stimulus induced movement and calculating RT**

Example of stimulus induced movement ("Twitch"), median accelerometer trace, and threshold for RT calculation. The threshold is 1/6 of the peak median acceleration as it was determined to the lowest possible threshold while not detecting the stimulus induced twitch as voluntary movement.

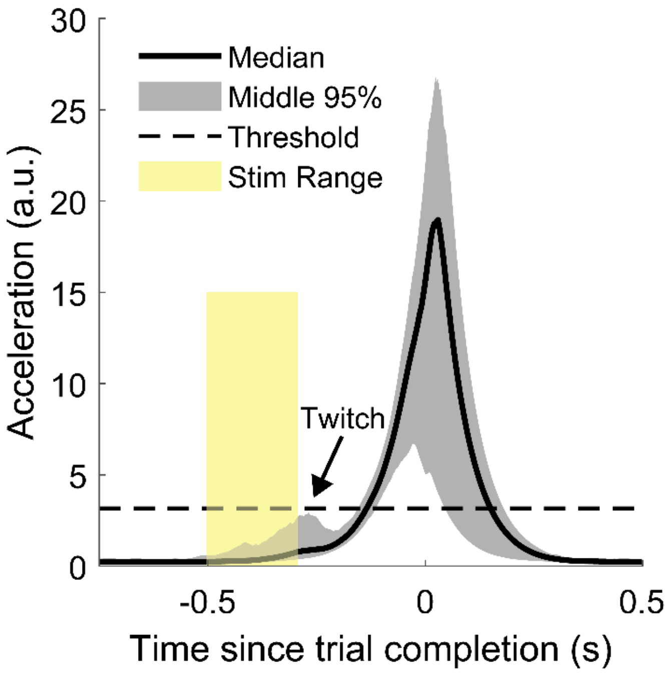

Supplementary Figure 4. Amplitude in Contralateral CS experiments

Instantaneous LFP band amplitude of the stimulated hemisphere for each epoch and stimulus timing during Contralateral CS experiments. Contralateral and ipsilateral refers to limb movement relative to the stimulated hemisphere.

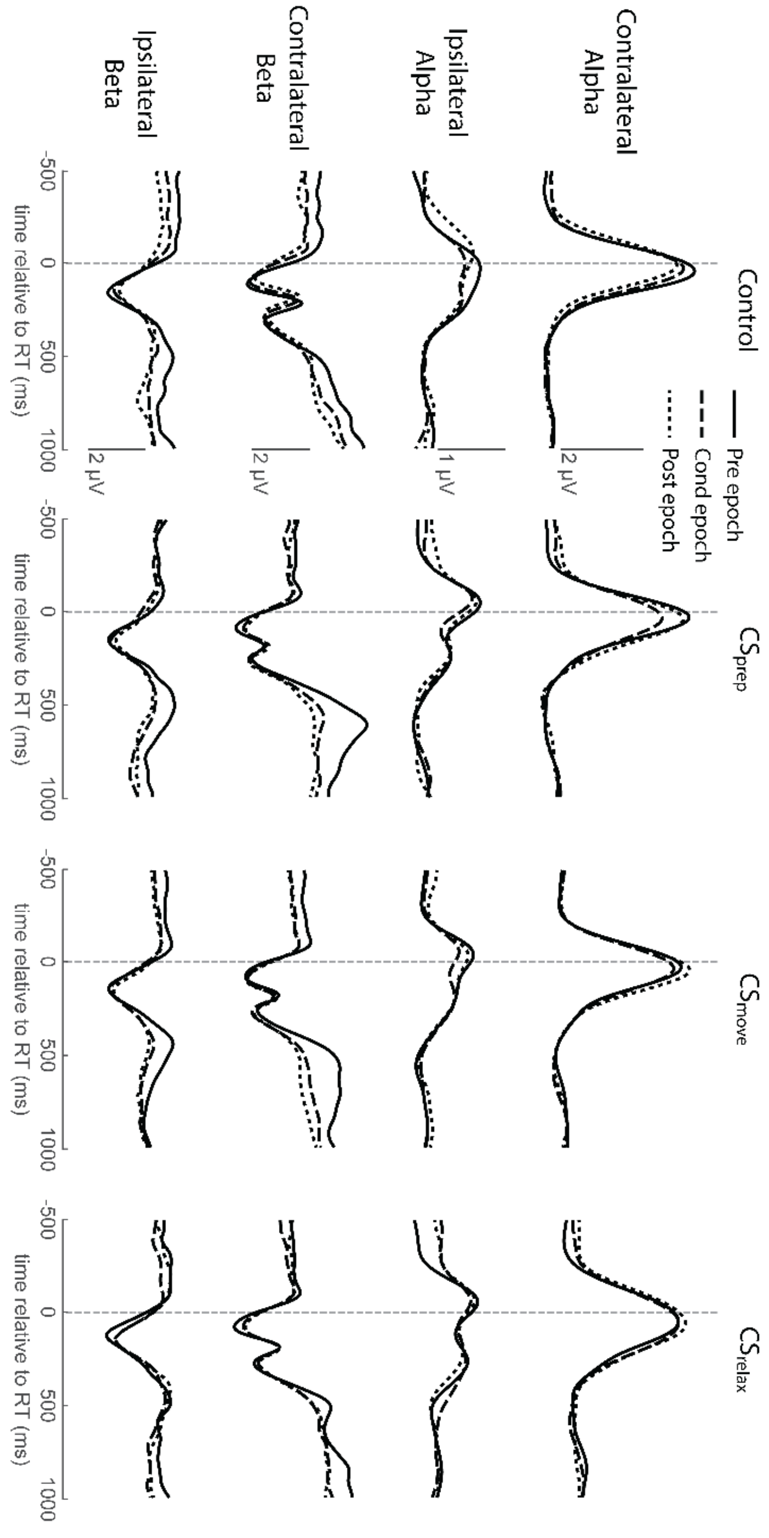

Supplementary Figure 5. Amplitude in Ipsilateral CS experiments

Instantaneous LFP band amplitude of the stimulated hemisphere for each epoch and stimulus timing during Ipsilateral CS experiments. Contralateral and ipsilateral refers to limb movement relative to the stimulated hemisphere.

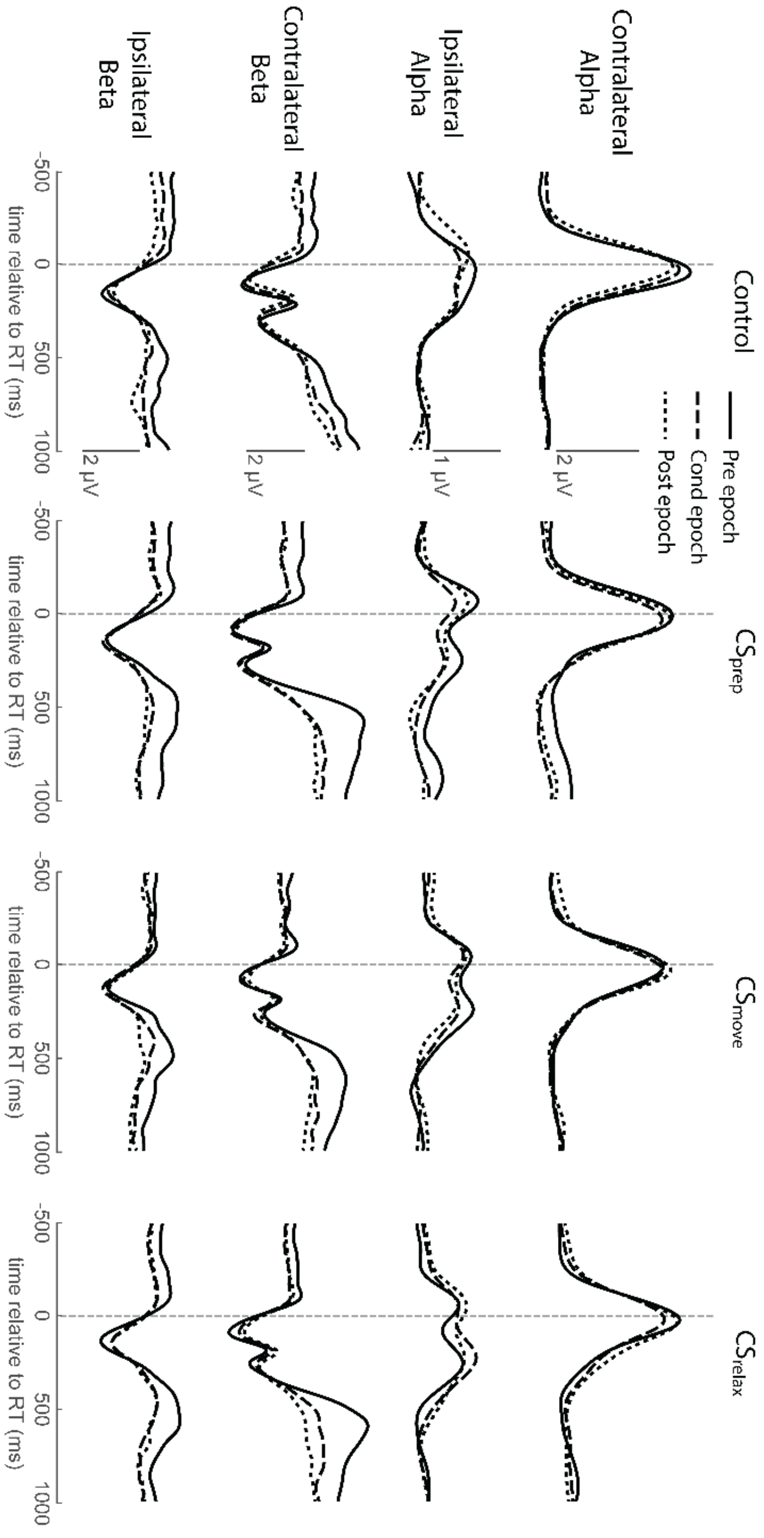

Supplementary Figure 6. Coherence in Contralateral CS experiments

Interhemispheric coherence for each epoch and stimulus timing during Contralateral CS experiments. Contralateral and ipsilateral refers to limb movement relative to the stimulated hemisphere.

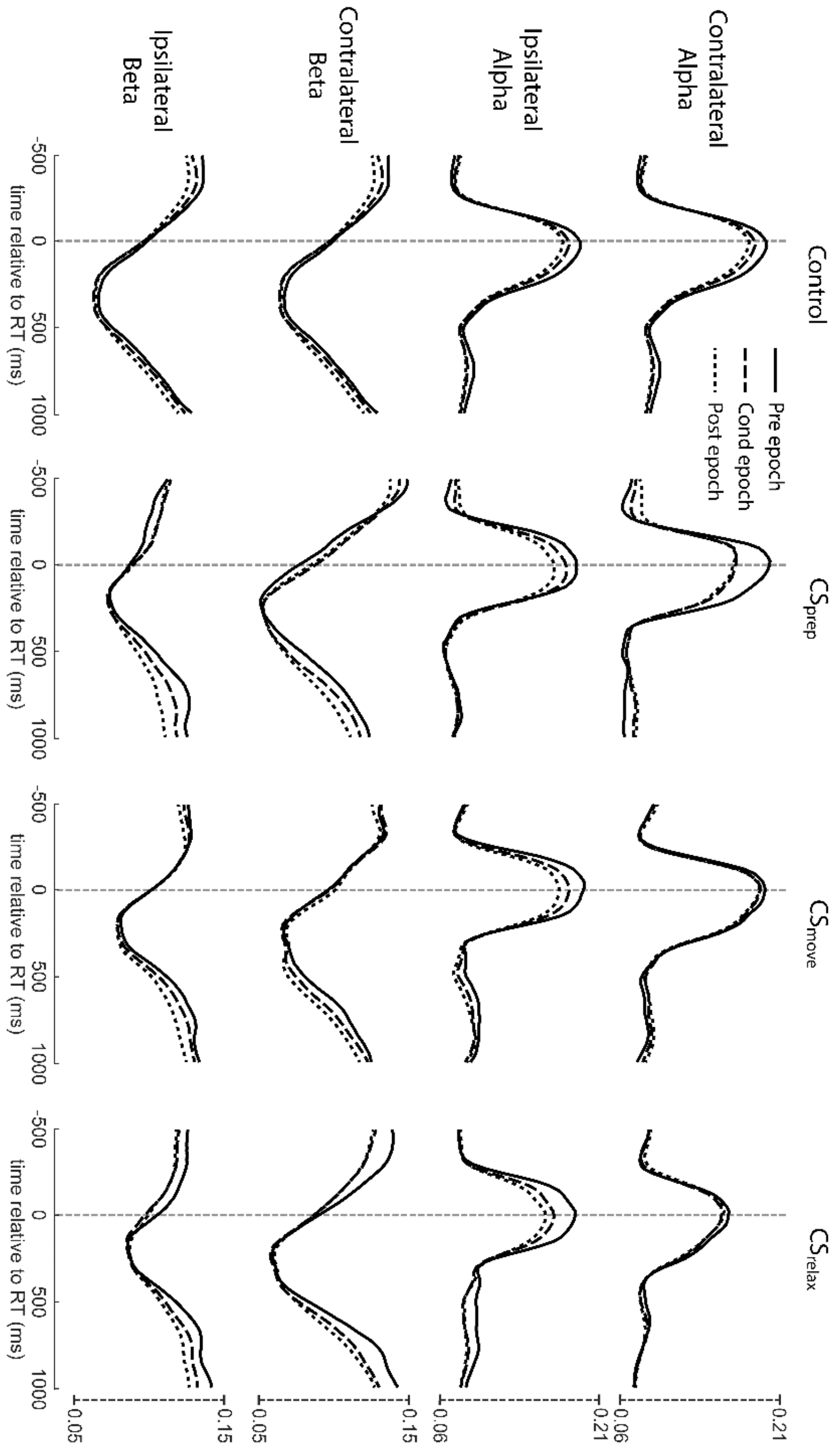

Supplementary Figure 7. Coherence in Ipsilateral CS experiments

Interhemispheric coherence for each epoch and stimulus timing during Ipsilateral CS experiments. Contralateral and ipsilateral refers to limb movement relative to the stimulated hemisphere.

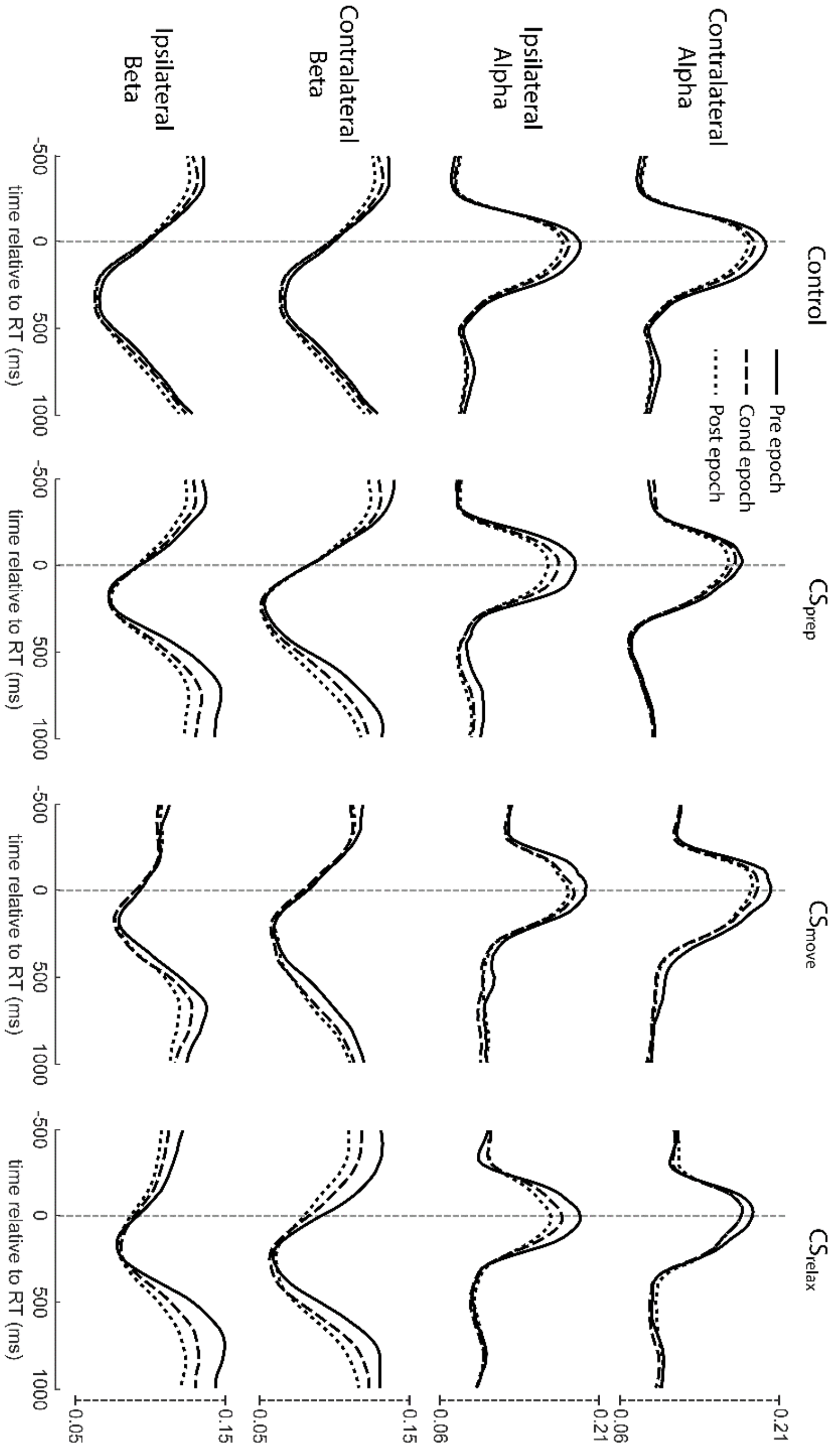

Supplementary Figure 8.   Changes in alpha coherence compared to changes in RT

Scatter plot showing the changes in alpha coherence plotted against the changes in RT for all experiment conditions and trial types with statistically significant changes in alpha. There is no statistically significant correlation between the two measures.

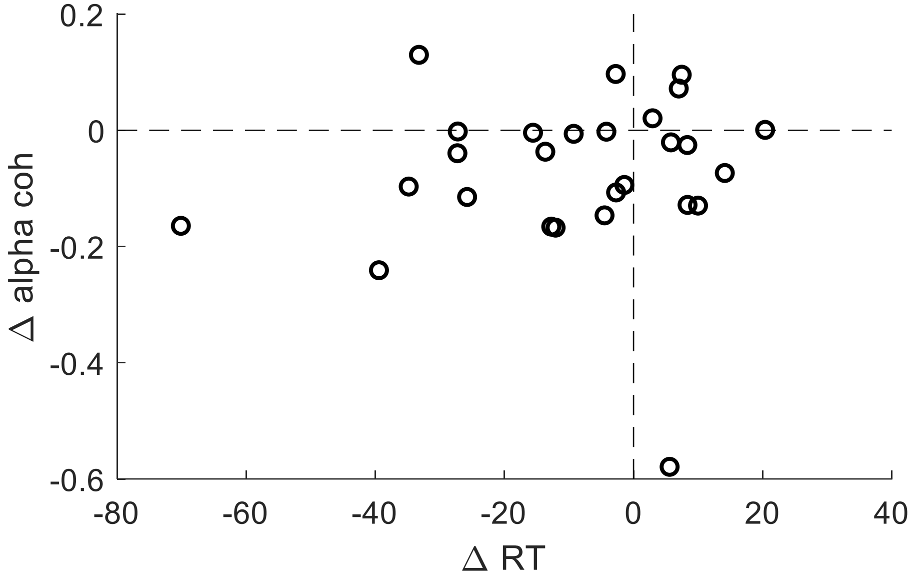

Supplementary Figure 9. Granger causality

Average pairwise Granger causality between all 32 channels of Monkey U during left and right trials of the Pre epoch and their significance across all conditioning experiments. 16 channels were in the left hemisphere (L channels) and 16 in the right hemisphere (R channels). Note the high causality and significance within hemispheres but sparse directionality between hemispheres.

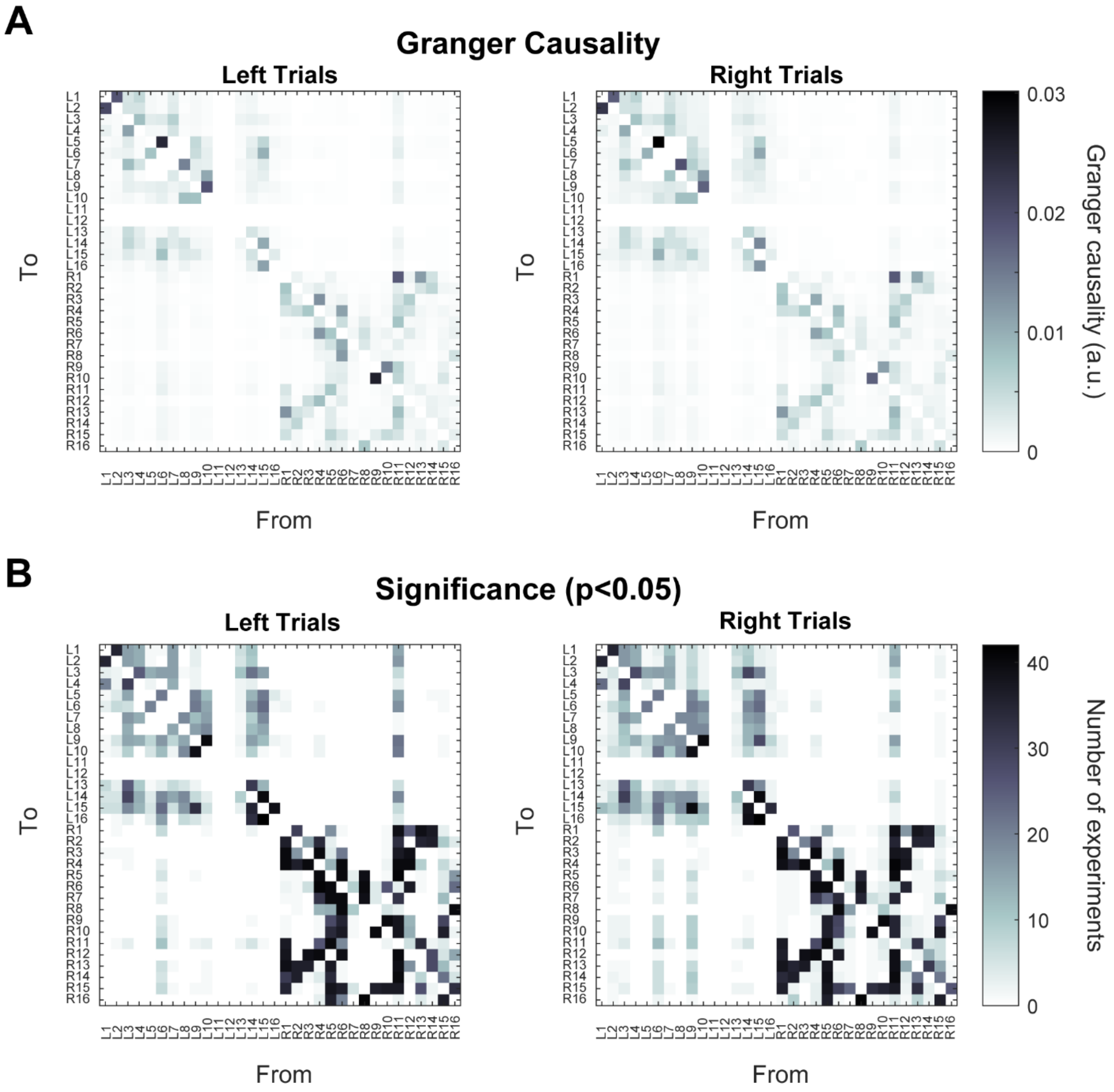

Supplement: Supplementary file 1 [file Data_Sheet_1.pdf]
